# Sodium Selenite Attenuates Balloon Injury-Induced and Monocrotaline-Induced Vascular Remodeling in Rats

**DOI:** 10.3389/fphar.2021.618493

**Published:** 2021-03-15

**Authors:** Changhong Cai, Yonghui Wu, Lebing Yang, Yijia Xiang, Ning Zhu, Huan Zhao, Wuming Hu, Lingchun Lv, Chunlai Zeng

**Affiliations:** ^1^ Department of Cardiology, Lishui Hospital of Zhejiang University, The Fifth Affiliated Hospital of Wenzhou Medical University, Lishui Municipal Central Hospital, Lishui, China; ^2^ Department of Cardiology, The Wenzhou Third Clinical Institute Affiliated To Wenzhou Medical University, Wenzhou People’s Hospital, Wenzhou, China

**Keywords:** vascular remodeling, vascular smooth muscle cells, sodium selenite, balloon injury, monocrotaline

## Abstract

Vascular remodeling (VR), induced by the massive proliferation and reduced apoptosis of vascular smooth muscle cells (VSMCs), is primarily responsible for many cardiovascular conditions, such as restenosis and pulmonary arterial hypertension. Sodium selenite (SSE) is an inorganic selenium, which can block proliferation and stimulate apoptosis of tumor cells; still, its protective effects on VR remains unknown. In this study, we established rat models with carotid artery balloon injury and monocrotaline induced pulmonary arterial hypertension and administered them SSE (0.25, 0.5, or 1 mg/kg/day) orally by feeding tube for 14 consecutive days. We found that SSE treatment greatly ameliorated the development of VR as evidenced by an improvement of its characteristic features, including elevation of the ratio of carotid artery intimal area to medial area, right ventricular hypertrophy, pulmonary arterial wall hypertrophy and right ventricular systolic pressure. Furthermore, PCNA and TUNEL staining of the arteries showed that SSE suppressed proliferation and enhanced apoptosis of VSMCs in both models. Compared with the untreated VR rats, lower expression of PCNA and CyclinD1, but higher levels of Cleaved Caspase-3 and Bax/Bcl-2 were observed in the SSE-treated rats. Moreover, the increased protein expression of MMP2, MMP9, p-AKT, p-ERK, p-GSK3β and β-catenin that occurred in the VR rats were significantly inhibited by SSE. Collectively, treatment with SSE remarkably attenuates the pathogenesis of VR, and this protection may be associated with the inhibition of AKT and ERK signaling and prevention of VSMC’s dysfunction. Our study suggest that SSE is a potential agent for treatment of VR-related diseases.

## Introduction

Vascular remodeling (VR) refers to adaptive modifications of the vascular wall structure and plays an essential part in the physiology of vascular homeostasis. On the other hand, pathological vascular remodeling is related to the origination and development of numerous cardiovascular conditions such as atherosclerosis, post-angioplasty restenosis and pulmonary arterial hypertension, which have the highest mortality rate in developing as well as developed countries (Sprague and Khalil, 2009; Becher et al., 2020). Vascular smooth muscle cells (VSMCs) constitute the major resident cells of the arteries. In response to vascular injury, VSMCs turn into a high rate of proliferation and reduce apoptosis, and move to the vessel’s luminal side, which is critical for remodeling processes in vascular disorders (Alexander and Owens, 2012; Bennett et al., 2016; Lacolley et al., 2017). Given the prominent role of VSMCs, identifying pharmacological interventions with combined anti-proliferation and pro-apoptosis properties is a vital step in the prevention and treatment of VR-related diseases.

Selenium (Se) is a crucial component of selenoproteins and an essential trace mineral in human bodies. Under standard physiological circumstances, a proportionately high amount of selenium in serum has a positive contribution to the function of the cardiovascular system, while a low amount of selenium in serum elevates the likelihood of cardiovascular diseases and mortality (Akbaraly et al., 2005; Dhanya et al., 2014). Sodium selenite (SSE) is the most common reagent that is used as a Se supplement for clinical use, which can inhibit tumor cell proliferation and migration as well as induce cell apoptosis (Okuno et al., 2014). In addition, SSE manages a spectrum of signals in tumor cells such as AKT, glycogen synthase kinase 3 beta (GSK3β)/β-catenin, extracellular-signal-regulated kinase (ERK), and phosphatase and tensin homologue deleted on chromosome 10 (PTEN), and because of these effects, SSE is a potential novel chemotherapy drug for cancer (Ren et al., 2009; Luo et al., 2013; Gong and Li, 2016; Kim et al., 2020).

Although the anti-proliferation and pro-apoptosis effects of SSE on cancer cells are well documented, the exact role and mechanism of SSE in VR-related diseases remains undetermined. In the present study, we evaluated the potential therapeutic effects of SSE on VSMC’s proliferation and apoptosis, as well as its impact on carotid and pulmonary artery remodeling caused by two different vascular injuries *in vivo*. To examine the mechanisms underlying its activity, we investigated the signaling pathways that are possibly influenced by SSE.

## Materials and Methods

### Animals and Reagents

Sprague-Dawley rats, male, were retrieved from the Experimental Animal Center of Zhejiang Province. The study protocol was authorized by the Ethics Review Board of Animal Use Application of The Lishui Municipal Central Hospital (Lishui, China) in compliance with the National Institutes of Health Guidelines For the Care and Use of Experimental Animals. Prior to the experiments, the rats were placed in a room under the controlled conditions of 20–26°C, 45–55% humidity, a 12 h light/dark cycle, and freely available water and standard rat chow.

SSE (purity>99.0%), bovine serum albumin (BSA) and monocrotaline (MCT) were obtained from Sigma Aldrich (Darmstadt, Germany). SSE was dissolved in normal saline. MCT was liquefied in 1M HCL neutralized with 1M NaOH, and thinned down with standard saline. Thereafter, PH was altered to 7.2–7.4. Anti-PCNA, anti-CyclinD1, anti-MMP2, anti-MMP9, anti-Bax, and anti-Bcl-2, antibodies were purchased from Abcam (Cambridge, United Kingdom). Anti-Cleaved Caspase-3, anti-p-GSK3β, anti-GSK3β, anti-β-catenin, anti-p-AKT, anti-AKT, anti-p-ERK, anti-ERK and anti-GAPDH were retrieved from Cell Signaling Technology (Danvers, MA, United States). The hematoxylin and eosin (H&E) stain kit was obtained from Solarbio (Beijing, China). The Colorimetric TUNEL Apoptosis assay kit was purchased from Beyotime Institute of Biotechnology (Jiangsu, China).

### Experimental Design

In the carotid artery balloon injury experiment, 48 rats weighing 350–400 g were separated into five different groups at random: I) the control group (n = 12) which underwent sham operation in the right carotid artery; II) the balloon injury (BI) group (n = 12) received BI in the left carotid artery; III) the SSE low group (n = 12) received BI in the left carotid artery and SSE at 0.25 mg/kg/day; IV) the SSE medium group (n = 12) received BI in the left carotid artery + SSE at 0.5 mg/kg/day; and V) the SSE high group (n = 12) received BI in the left carotid artery + SSE at 1 mg/kg/day. This high dose was chosen in accordance with data contained in the literature (Jacobs and Forst, 1981; Laureano-Melo et al., 2015).

In the pulmonary arterial hypertension experiment, 48 rats weighing 220–240 g were separated into five different groups at random: I) the control group which received standard saline (n = 8); II) the MCT group received MCT at 60 mg/kg (n = 10); III) the SSE low group received MCT and SSE at 0.25 mg/kg/day (n = 10); IV) the SSE medium group received MCT and SSE at 0.5 mg/kg/day (n = 10); and V) the SSE high group received MCT and SSE at 1 mg/kg/day (n = 10).

### Rat Carotid Artery Balloon Injury Model

At day 0, the rats were sedated, intraperitoneally, with sodium pentobarbital (30 mg/kg). After that, to uncover the left external carotid artery, a mid-line incision in the neck was performed. Through an arteriotomy, a 2.0 Fogarty arterial embolectomy catheter was inserted into the artery and moved into the common carotid artery. The balloon was first blown up with 2 atm and thereafter removed in a rotating movement in the direction of the external carotid artery. The routine was repeated thrice. After removal of the catheter, ligation of the external carotid artery was performed. In sham operated rats, the right common carotid artery was exposed without catheterization. Two weeks afterward, the rats of the SSE groups received intragastrically varying dosages of SSE, which was continued every day for the duration of 2 weeks.

Following these four weeks, the rats were euthanized and the injured carotid arteries were gathered for histology and western blot analyses.

### Rat Pulmonary Arterial Hypertension Model

The pulmonary arterial hypertension model was created through a single subcutaneous injection of MCT per 60 mg/kg on day 0. Two weeks afterward, the rats of the SSE groups received intragastrically varying dosages of SSE, which was continued every day for the duration of two weeks. Four weeks following the MCT injection, anesthesia of the rats with sodium pentobarbital (30 mg/kg) was conducted intraperitoneally. Thereafter, measurement of the right ventricle systolic pressure (RVSP) was performed according to previously reported literature (Zhu et al., 2016). After the hemodynamic measurements, separation of the right ventricle (RV) was conducted, and the weight proportion of RV to left ventricle (LV) plus septum (S), and the weight proportion of RV to body weight (BW) were computed. Then, the tissues of the lung were removed for histology and western blot analyses.

### Histological and Morphometric Analysis

The isolated carotid arteries and lungs taken from the rats in all experimental groups were fixated in 4% paraformaldehyde and cut into 4 μm tissue sections. H&E staining was conducted in accordance with the manufacturer’s protocols. The images were obtained through a light microscope (Nikon Corporation). The morphometric analysis was performed with the Image-Pro Plus image analysis software (Media Cybernetics, Inc., Rockville, MD, United States).

Intimal hyperplasia was defined as the formation of a neointimal layer medial to the internal elastic lamina. Intima area (IA) = internal elastic lamina area − luminal area and the medial area (MA) = external elastic lamina area − internal elastic lamina area. Thereafter, the intima/media (I/M) ratio was calculated.

For each pulmonary artery with diameters from 50 to 150μm, the degree of wall thickness (WT) was calculated in the following ways: the fraction of vascular wall thickness as WT% = 100% × (external diameter − internal diameter)/external diameter and the fraction of the vascular wall area as WA% = 100% × (total vascular area − lumen area)/total vascular area. For each section, three vessels were randomly selected for measurement. Two independent lung sections were analyzed for each animal, and the resulting values were averaged.

### Immunohistochemistry

Carotid artery and lung sections were dewaxed and rehydrated. Then, at 100°C antigens were retrieved and blocked at room temperature with 5% BSA for 1 h. Following incubation overnight of the sections with anti-PCNA antibodies at 4°C, they were incubated with the addition of corresponding secondary antibodies. Then, the sections were visualized at room temperature with DAB staining for 5 min and counterstained, also at room temperature, with hematoxylin for 2 min. TUNEL assay was used to analyze apoptosis according to instructions of the manufacturer. To quantitatively calculate the percentage of PCNA and TUNEL positive cells (positive cells% = 100% × number of positive cell nuclei/total number of cell nuclei), the number of normal and positive cell nuclei was counted in three fields of each section.

### Western Blot

Proteins were extracted from the carotid arteries (two or three blood vessels merged into one sample) and lungs using lysis buffer containing protease and phosphatase inhibitors. After complete homogenization on ice, the samples were centrifuged. Then the supernatants were separated with 10–12% SDS PAGE and relocated to PVDF membranes. Subsequently, the membranes were blocked at room temperature with 5% BSA for 2 h and incubated together with primary antibodies at 4°C overnight. Then, the membranes were washed and incubated at room temperature with the secondary antibodies, anti-rabbit, or anti-mouse, for 1 h. Chemiluminescence was used to visualize the protein bands. Through densitometric quantification with AlphaView software (ProteinSimple, San Jose, CA, United States), the protein content was analyzed and GAPDH was used as an internal control. Densitometry of western blot was expressed relative to control groups.

### Statistical Analysis

Analyses were conducted with GraphPad Prism version 5.0 (GraphPad Software, Inc., La Jolla, CA, United States) and the data were depicted as the mean and standard error of the mean. The differences among multiple groups were analyzed with one-way ANOVA followed by the Student-Newman-Keuls post-hoc test. A *p*-value below 0.05 was regarded as an indication of statistical significance.

## Results

### SSE Attenuated Neointimal Formation Induced by Carotid Artery Balloon Injury

**FIGURE 1:**
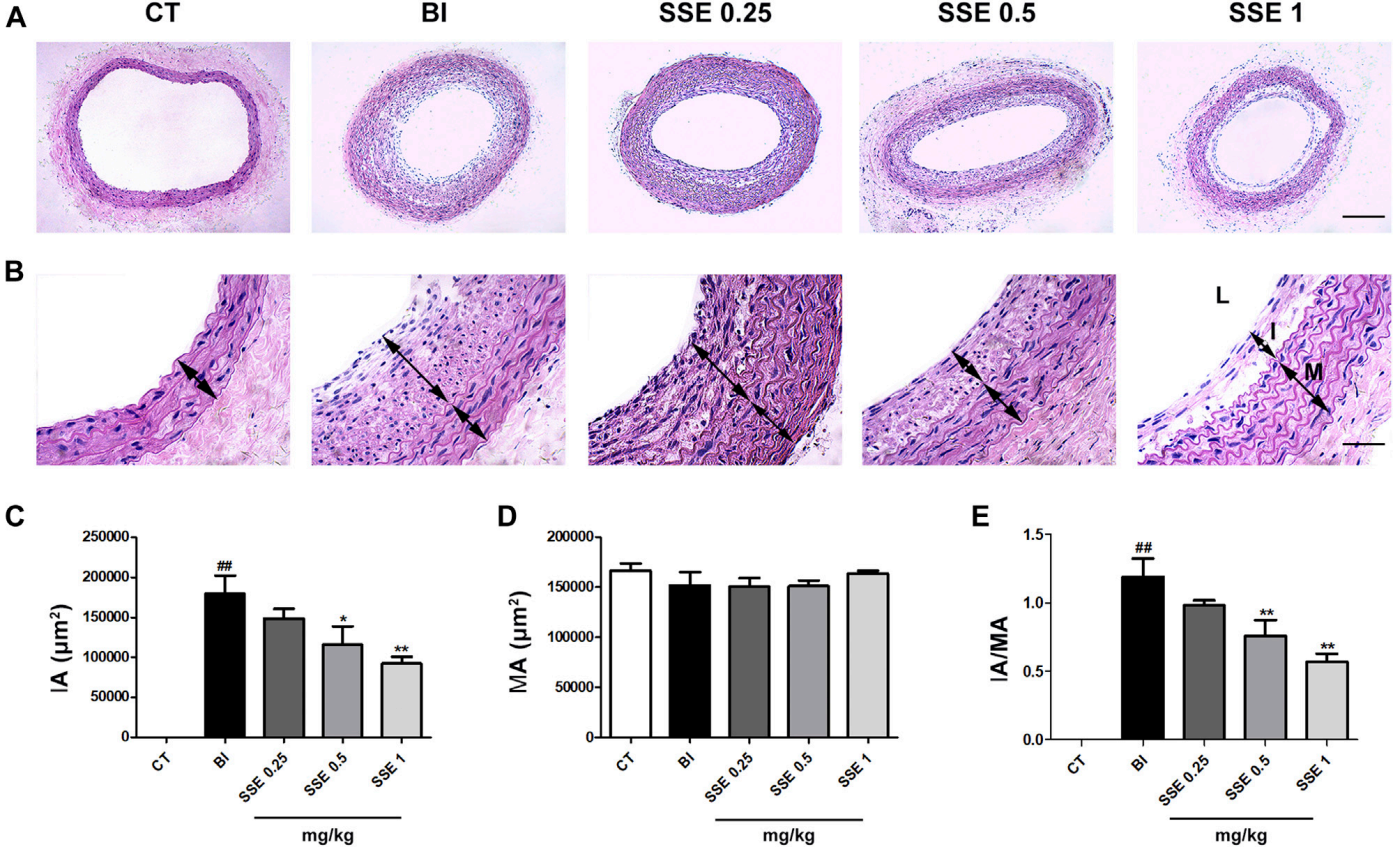
SSE attenuated neointimal formation induced by carotid artery balloon injury. **(A)**: Representation of neointimal formation in arteries with H&E staining. Magnification ×100, scale bar: 100 μm. **(B)**: Representation of neointimal formation in arteries with H&E staining. L: lumen, I: intima, M: media. Magnification ×400, scale bar: 50 μm. **(C)**, **(D)** and **(E)**: Quantitative analysis for the values of IA, MA and the IA/MA ratio from images. n = 4–5 per group. ##*p* < 0.01 vs. CT group, *p < 0.05, **p < 0.01 vs. BI group. CT: control, BI: balloon injury, SSE0.25: sodium selenite 0.25 mg/kg, SSE0.5: sodium selenite 0.5 mg/kg, SSE1: sodium selenite 1 mg/kg, IA: intimal area, MA: medial area.

Vascular histomorphometric analysis revealed that the intima in BI rats was thickened and that the luminal area was significantly narrowed in comparison to the control group. Notably, we observed that SSE concentrations of both 0.5 and 1 mg/kg markedly attenuated neointimal formation and stenosis, as indicated by the dramatically reduced intimal area (IA) and I/M ratio in the injured vessels (Figure 1).

### SSE Attenuated Monocrotaline Induced Pulmonary Arterial Hypertension

**FIGURE 2:**
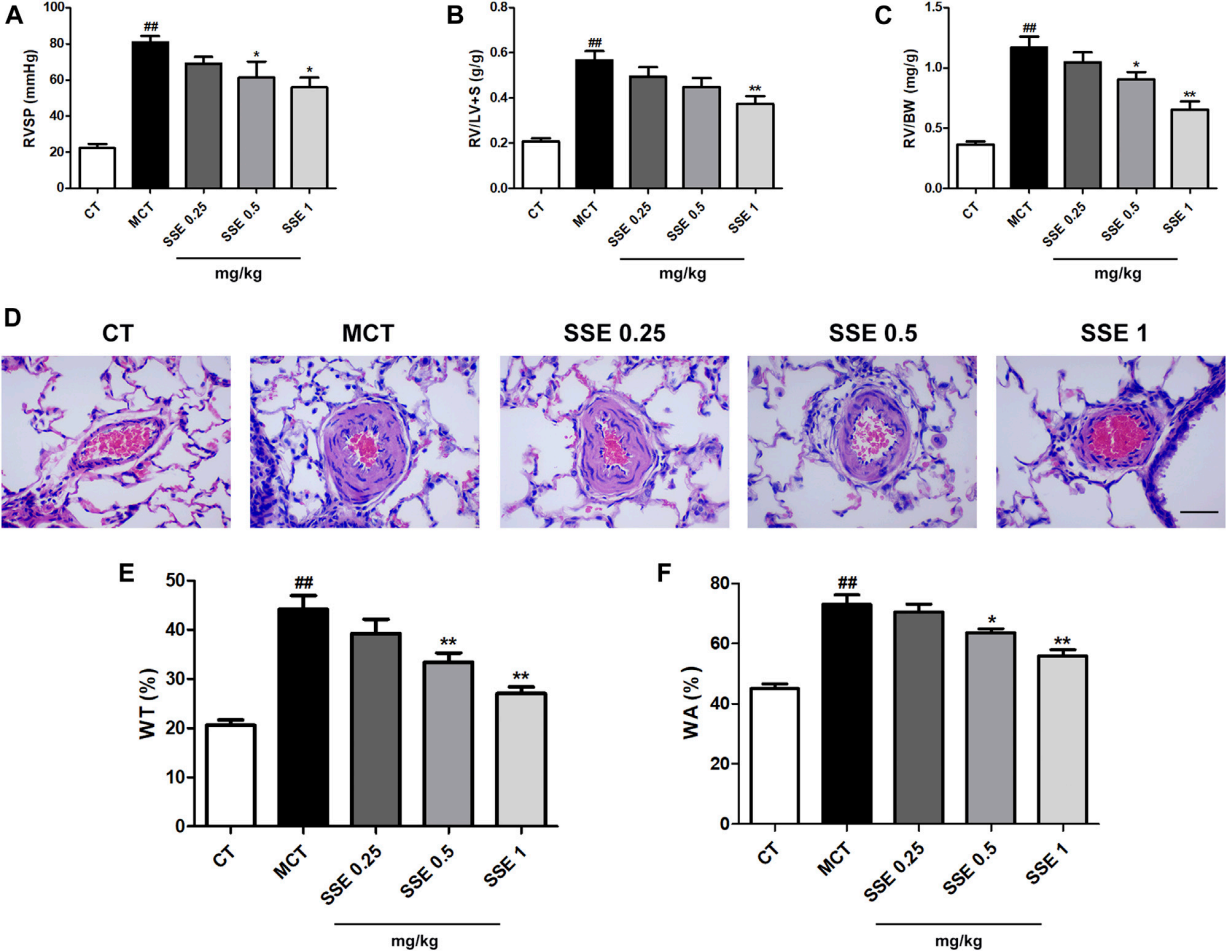
SSE attenuated pulmonary arterial hypertension induced by MCT. **(A)**, **(B)** and **(C)**: Quantitative analysis for the values of RVSP, RV/LV + S and RV/BW. n = 6–8 per group. **(D)**: Representation of pulmonary artery remodeling identified by H&E staining. Magnification ×400, scale bar: 50 μm. **(E)** and **(F)**: Quantification analysis of SSE on WT% and WA% for the pulmonary artery. n = 6. ##*p* < 0.01 vs. CT group, *p < 0.05, **p < 0.01 vs. MCT group. RVSP: right ventricular systolic pressure, RV/LV + S: right ventricle/left ventricle plus septum weight ratio, RV/BW: right ventricle/body weight ratio. WT: wall thickness, WA: wall area.

The parameters RVSP, right ventricle/left ventricle plus septum weight ratio (RV/LV + S) and right ventricle/body weight ratio (RV/BW) that reflect the severity of pulmonary arterial hypertension, were significantly increased after MCT injection, whereas reduction followed in the SSE 1 mg/kg group (Figures 2A–C). In addition, MCT treated rats exhibited obvious increased pulmonary artery wall thickness compared to the control group. Furthermore, SSE treatment significantly inhibited these pathological alternations in a dose dependent manner (Figures 2D–F). Overall, the results demonstrated that SSE was effective in inhibiting pulmonary arterial hypertension.

### SSE Inhibits the Proliferation of VSMCs

**FIGURE 3:**
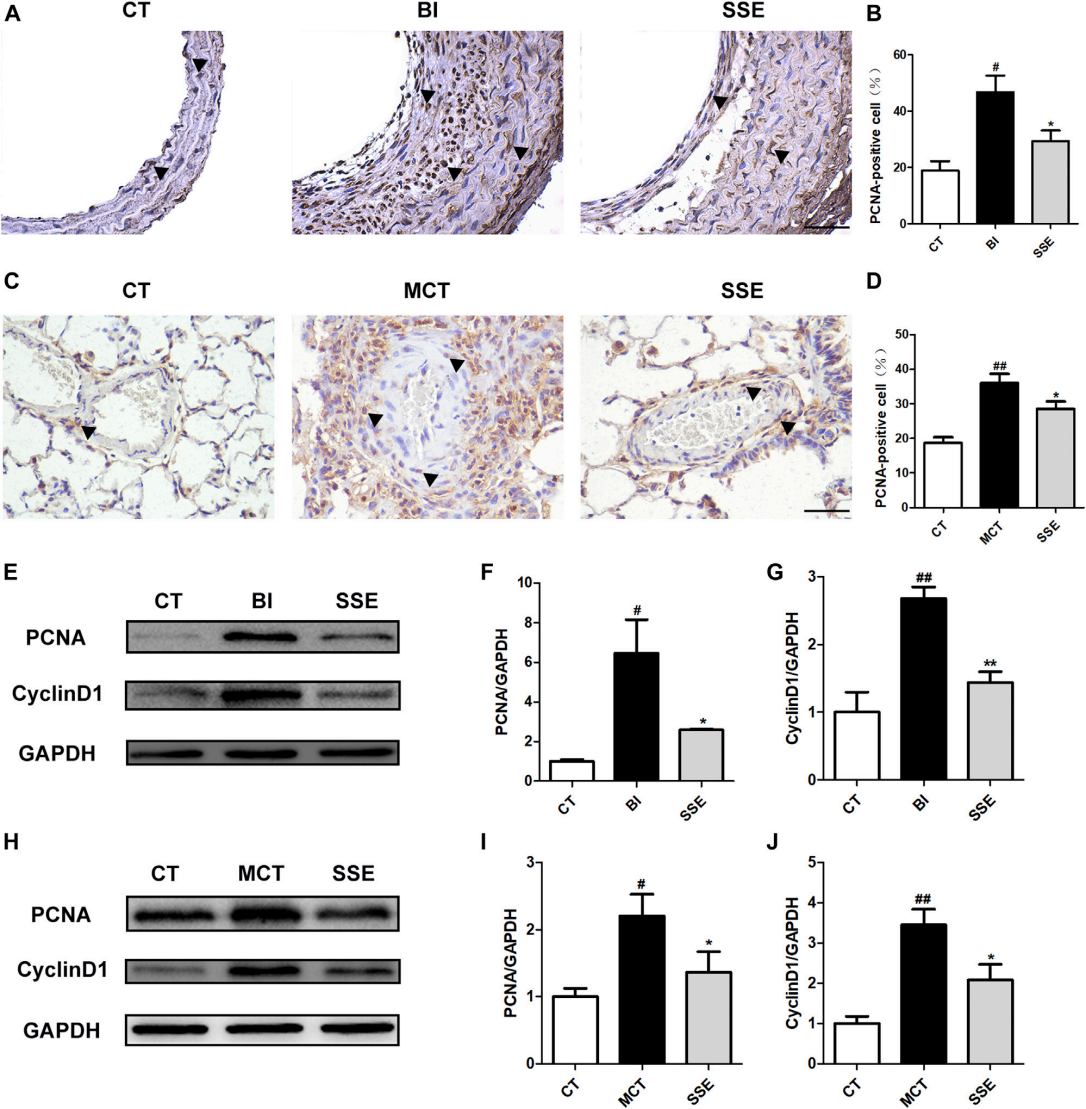
SSE inhibited the proliferation of VSMCs. Representative photomicrographs of serial carotid artery **(A)** and lung **(C)** sections were immunostained for PCNA. Magnification ×400, scale bars = 50 μm. In the images, the black arrows point out the positive cells. The bar graph reflects the percentage of PCNA-positive cells in carotid arteries (**B**, n = 3 per group) and pulmonary arteries (**D**, n = 6 per group). The expression of PCNA and CyclinD1 in the carotid arteries (**E,F,G**, n = 3 per group) and lungs (**H,I,J**, n = 6 per group) was examined by western blot analysis. #*p* < 0.05, ##*p* < 0.01 vs. CT group, *p < 0.05, **p < 0.01 vs. BI/MCT group.

A high-dose treatment of SSE (1 mg/kg) was selected for further analyses to additionally clarify the potential mechanisms responsible for the protective effects on VR. Considering that VSMCs’ proliferation is one of the most direct causes of VR, the cellular proliferation markers PCNA and CyclinD1 were detected and compared in balloon injury-stimulated carotid arteries and MCT-stimulated lungs from different groups. The control group demonstrated very few PCNA-positive brown cells in the blood vessels’ media, while the rats that received balloon injury and MCT injection showed a great number of PCNA-positive cells in the media and intima (the cells that were positive in balloon injury rats were primarily found in the intima). Notably, SSE significantly decreased the positive rate (Figures 3A–D). The suppression of SSE in proliferation was further validated using western blot analysis. As Figures 3E–J shows, PCNA and CyclinD1 proteins’ expression levels were obviously elevated in both balloon injury and MCT treated rats, whereas the opposite effect was observed in SSE rats.

### SSE Enhanced the Apoptosis of VSMCs

**FIGURE 4:**
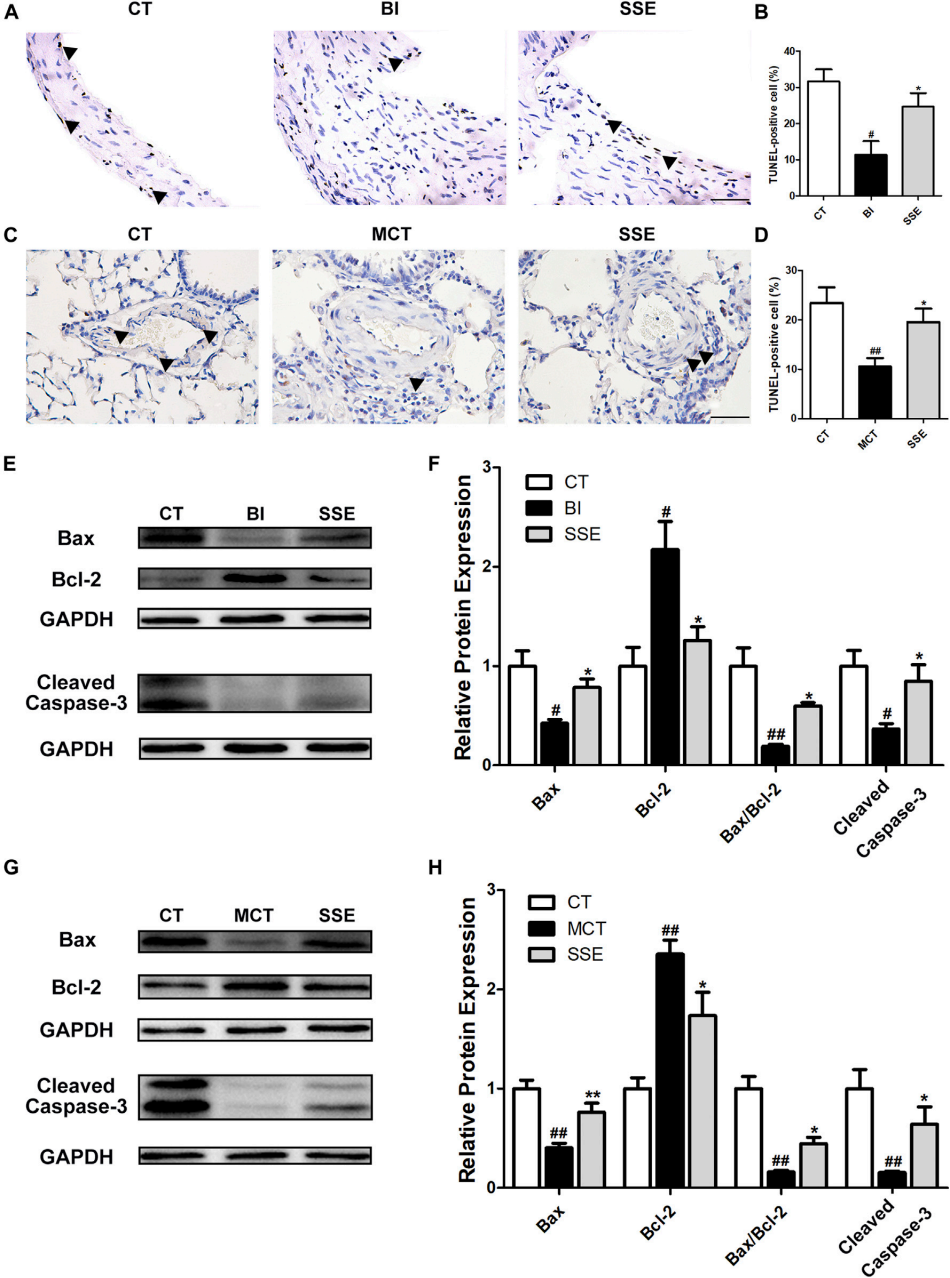
SSE enhanced the apoptosis of VSMCs. Representative photomicrographs of serial carotid artery **(A)** and lung **(C)** sections were immunostained for TUNEL. Magnification ×400, scale bars = 50 μm. In the images, the black arrows point out the positive cells. The bar graph reflects the percentage of TUNEL-positive cells in carotid arteries (**B**, n = 3 per group) and lungs (**D**, n = 6 per group). The expression of Bax, Bcl-2 and Cleaved Caspase-3 in carotid arteries (**E,F**, n = 3 per group) and lungs (**G,H**, n = 6 per group) was examined by western blot analysis. #*p* < 0.05, ##*p* < 0.01 vs. CT group, *p < 0.05, **p < 0.01 vs. BI/MCT group.

To confirm the effects of SSE on apoptosis of VSMCs, the carotid arteries and lungs of rats were subjected to TUNEL staining, and the percentage of apoptosis cells were calculated. As shown in Figures 4A–D, balloon injury and MCT markedly attenuated VSMCs’ apoptosis, which was further enhanced by SSE. Moreover, SSE significantly decreased the protein Bcl-2, which is anti-apoptotic, while it further increased the proteins Bax and Cleaved Caspase-3, which are pro-apoptotic (Figures 4E–H). These results indicate that SSE increased the apoptosis of VSMCs caused by balloon injury and MCT.

### SSE Alleviated the Expression of Matrix Metalloproteinases

**FIGURE 5:**
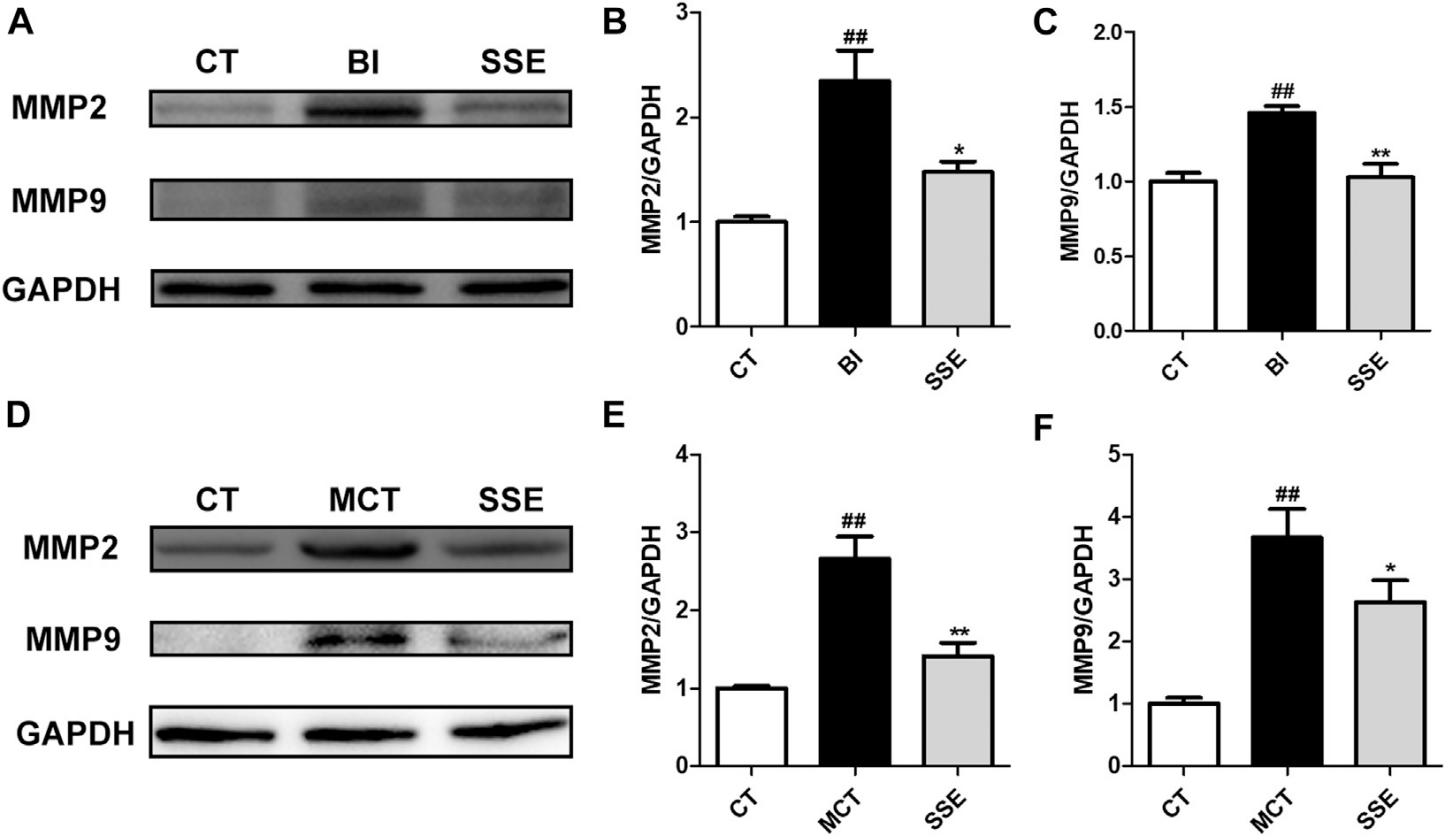
SSE alleviated the expression of matrix metalloproteinases. The expression of MMP2 and MMP9 in carotid arteries (**A,B,C**, n = 3 per group) and lungs (**D,E,F**, n = 6 per group) were examined by western blot analysis. #*p* < 0.05, ##*p* < 0.01 vs. CT group, *p < 0.05, **p < 0.01 vs. BI/MCT group.

During VR, VSMCs clearly show migratory properties. Matrix metalloproteinases (MMPs), especially MMP2 and MMP9, are largely involved in and profoundly mediate the migration of VSMCs. Therefore, we examined the protein expression levels of MMP2 and MMP9 in injury induced carotid arteries and MCT treated lungs. Western blot analysis revealed significantly up-regulated MMP2 and MMP9 expression post-injury, which was dramatically alleviated by SSE treatment (Figure 5).

### SSE Suppressed Activation of the AKT and ERK Signaling Pathways

**FIGURE 6:**
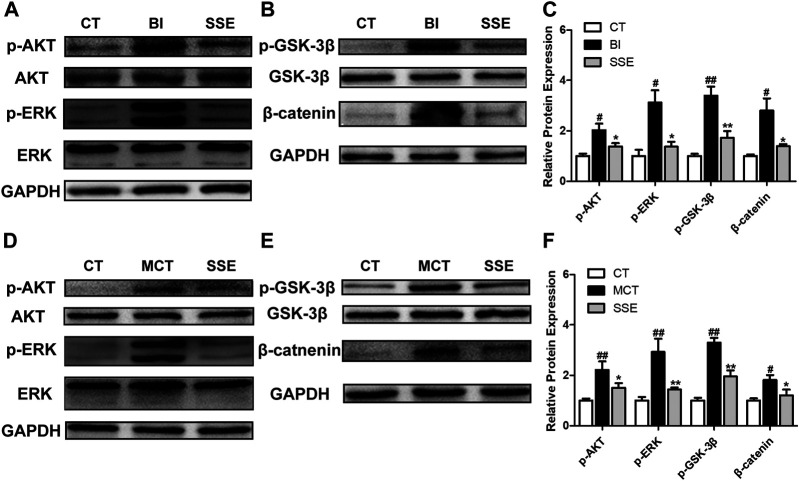
SSE suppressed activation of the AKT and ERK pathways. The expressions of p-AKT, AKT, p-ERK, ERK, p-GSK3β, GSK3β, and β-catenin in carotid arteries (**A,B,C**, n = 3 per group) and lungs (**D,E,F**, n = 6 per group) were analyzed by western blot analysis. #*p* < 0.05, ##*p* < 0.01 vs. CT group, *p < 0.05, **p < 0.01 vs. BI/MCT group.

Vascular injury that triggers AKT and ERK signaling activation induces a crucial modification in the process of arterial reconstruction. Western blot demonstrated that p-AKT/AKT, p-ERK/ERK, p-GSK3β/GSK3β and β-catenin in balloon injury-induced rats were significantly up-regulated compared with the control rats, however, these increases were diminished by SSE (Figures 6A–C). Meanwhile, similar results were observed in lungs isolated from SSE treated rats in response to MCT (Figures 6D–F).

## Discussion

Selenium is an essential trace element found in mammals (Rayman et al., 2008). The main forms of selenium are divided into organic selenium [such as CH_3_SeH and seleno-L-methionine (SeMet)] and inorganic selenium [such as SSE (Na_2_SeO_3_)] (Okuno et al., 2014). Previous studies have shown that SSE regulates multiple signaling pathways and could potentially be used clinically for systemic conditions such as different forms of cancers, inflammation, and bacterial infections (Pfister et al., 2016; Amoolya et al., 2018; Soliman et al., 2018; Wu et al., 2019). In this study, we demonstrate for the first time that SSE reversed the progression of VR induced by carotid artery balloon injury and MCT injection in rats. In both models, SSE suppressed proliferation but promoted apoptosis of VSMCs *in vivo*, indicating that SSE might be a promising drug for VR-related diseases.

Obstruction of the coronary flow inducted by atherosclerotic diseases can be attenuated by stent implantation. Nonetheless, the damage to the tunica media leads to restenosis and massive neointimal formation, which results in recurring ischemia (Stone et al., 2008). Therefore, it is necessary to design new drug eluting stents (Cheng et al., 2017). In the research of vascular restenosis postoperatively, a broad range of different animal models have been utilized. Although, until now, *in vivo* balloon injury rat models are still the most commonly utilized models, since their capacity to replicate neointimal formation and its pathogenesis are similar to humans (Orozco et al., 2012; Holt and Tulis, 2013). Therefore, our study used this model and confirmed that SSE exhibited, in rats, protection against balloon injury-induced neointimal formation, and this effect was dose dependent.

Pulmonary arterial hypertension is also a common but devastating cardiovascular disease based on VR (Thompson and Lawrie, 2017). In the present study, we used the previously described method to establish a pulmonary arterial hypertension model (Wu et al., 2020) and observed that the rats, which received daily SSE treatments two weeks subsequent to the MCT injection, had large decreases in right ventricular hypertrophy, pulmonary arterial remodeling, and pulmonary artery pressure compared with only MCT injected rats. Collectively, these results show the suppressive effect of SSE on VR-related diseases.

In vascular homeostasis, alteration of VSMC’s phenotype is fundamental. When blood vessels endure severe damage, VSMCs will change into a synthetic state from a contractile state, in which their proliferative and apoptosis resistance capacities are enhanced, and remodeling occurs gradually (Rensen et al., 2007). Proliferating cell nuclear antigen (PCNA), which is in VSMCs used as a proliferation marker, has been confirmed to be related closely to the cellular DNA’s production; therefore, the level of cell proliferation can be indicated by the amount of PCNA expression (Xu et al., 2015). The present study demonstrated that SSE inhibited the increase of, both the balloon injury and MCT injection induced, PCNA positive cells. Furthermore, previous studies have demonstrated the effectivity of SSE in eliminating cancerous cells and its close relation to the induction of apoptosis (Shigemi et al., 2017; Tao et al., 2018). In line with anti-cancer effects, SSE attenuated apoptosis resistance in VSMCs, as evidenced by growth in the number of TUNEL positive cells and the expression of pro-apoptosis markers Bax/Bcl-2 and Cleaved Caspase-3.

VSMCs maintain the capacity to differentiate unlike cardiac muscle cells, skeletal cells and other type of cells. VSMCs have the ability to transdifferentiate into a phenotype that is synthetic by secreting MMPs (mainly MMP2 and MMP9) and increasing extracellular matrix (ECM) deposition. Furthermore, the deposition of ECM promotes VSMC’s proliferation and migration (Kim et al., 2013; Kim and Ko, 2014). In our study, SSE decreased expression of MMP2 and MMP9 in the carotid artery in the balloon injury model, as well as the lung in the pulmonary arterial hypertension model.

The dynamic process of VR involves numerous molecular signaling cascades governing pathological growth and phenotypic switching of VSMCs (Rabinovitch, 2012; Jochen et al., 2015; Wu et al., 2016). Mounting evidence have shown that the activity of AKT and ERK is significantly enhanced in rat models of VR induced by balloon injury and MCT and vascular disease patients (Muto et al., 2007; Xu et al., 2013; Cai et al., 2019). The up-regulated AKT and ERK subsequently promote the proliferation, migration and apoptosis resistance of VSMCs by activating phosphorylated GSK3β (Ser9) to reduce β-catenin degradation (Riascos-Bernal et al., 2017; Li et al., 2019). Therefore, inhibition of AKT and ERK signaling is closely related to the maintenance of vascular homeostasis. Our data have shown that, in the balloon injured carotid arteries as well as the MCT treated lungs, SSE treatment lead to a reduction of the phosphorylated AKT, ERK, GSK3β (Ser9), and β-catenin, indicating that SSE significantly inhibited the AKT and ERK pathways.

Based on results from the present study, balloon injury and MCT injection induced the increase in PCNA, CyclinD1 proteins and decrease in Bax/Bcl-2, Cleaved Caspase-3. The changes of these proteins indicate that vascular injury caused by balloon injury and MCT leads to excessive proliferation and reduced apoptosis of VSMCs, and eventually to VR (Rensen et al., 2007). Therefore, we proposed that SSE improves VSMCs dysfunction and subsequently alleviates VR, and this process may be mediated by suppressing the AKT and ERK signals.

It has been reported that SSE inhibits tumor growth by regulating GSK3β/β-catenin and its upstream AKT and ERK signals (Ren et al., 2009; Kim et al., 2020). We speculate that SSE may suppress the activation of GSK3β-β/catenin by affecting AKT, ERK or their crosstalk, thus restore VSMCs to normal. In addition, the phenotype of VSMCs is strictly controlled by the platelet-derived growth factor (PDGF). Both AKT and ERK are crucial intermediate molecules in the signal transduction process of PDGF regulating VSMCs (Zhu et al., 2019). Perhaps, SSE has a certain antagonistic effect on PDGF. In order to further clarify the mechanism of action of SSE, relevant *in vitro* cell experiments need be performed in the future.

In conclusion, in the present study, using both rat carotid artery balloon injury model and MCT induced pulmonary arterial hypertension model, an inorganic selenium, SSE, was recognized as a prospective agent of VR according to its mediatory effects on the proliferation and apoptosis of VSMCs. The ability of SSE to alleviate VR may depend on its inhibition of AKT and ERK signaling. Even though further research is needed, the current study contributes to the standpoint that SSE is a promising candidate for the treatment of VR-related diseases.

## Data Availability

The original contributions presented in the study are included in the article/Supplementary Material, further inquiries can be directed to the corresponding author.

## References

[B1] Akbaraly N. T. Arnaud J. Hininger-Favier I. Gourlet V. Roussel A. M. Berr C. (2005). Selenium and mortality in the elderly: results from the EVA study. Clin. Chem. 51 (11), 2117–2123. 10.1373/clinchem.2005.055301 16123147

[B2] Alexander M. R. Owens G. K. (2012). Epigenetic control of smooth muscle cell differentiation and phenotypic switching in vascular development and disease. Annu. Rev. Physiol. 74 (1), 13–40. 10.1146/annurev-physiol-012110-142315 22017177

[B3] Amoolya N. Meera N. Muhammed M. Mary A. (2018). Inhibition and inactivation of uropathogenic *Escherichia coli* biofilms on urinary catheters by sodium selenite. Int. J. Mol. Sci. 19 (6), 1703. 10.3390/ijms19061703 PMC6032314 29880781

[B4] Becher T. Riascos-Bernal D. F. Kramer D. J. Almonte V. Chi J. Tong T. (2020). Three-dimensional imaging provides detailed atherosclerotic plaque morphology and reveals angiogenesis after carotid artery ligation. Circ. Res. 126 (5), 619–632. 10.1161/CIRCRESAHA.119.315804 31914850 PMC7047629

[B5] Bennett M. R. Sinha S. Owens G. K. (2016). Vascular smooth muscle cells in atherosclerosis. Circ. Res. 118 (4), 692–702. 10.1161/CIRCRESAHA.115.306361 26892967 PMC4762053

[B6] Cai C. Xiang Y. Wu Y. Zhu N. Zhao H. Xu J. (2019). Formononetin attenuates monocrotalineinduced—pulmonary arterial hypertension via inhibiting pulmonary vascular remodeling in rats. Mol. Med. Rep. 20 (6), 4984–4992. 10.3892/mmr.2019.10781 31702810 PMC6854580

[B7] Cheng W. L. She Z. G. Qin J. J. Guo J. H. Gong F. H. Zhang P. (2017). Interferon regulatory factor 4 inhibits neointima formation by engaging Krüppel-like factor 4 signaling. Circulation 136 (15), 1412–1433. 10.1161/CIRCULATIONAHA.116.026046 28851732

[B8] Dhanya B. L. Swathy R. P. Indira M. (2014). Selenium downregulates oxidative stress-induced activation of leukotriene pathway in experimental rats with diabetic cardiac hypertrophy. Biol. Trace Elem. Res. 161 (1), 107–115. 10.1007/s12011-014-0076-7 25062888

[B9] Gong J. Li L. (2016). Sodium selenite inhibits proliferation of gastric cancer cells by inducing SBP1 expression. Tohoku J. Exp. Med. 239 (4), 279–285. 10.1620/tjem.239.279 27477809

[B10] Holt A. W. Tulis D. A. (2013). Experimental rat and mouse carotid artery surgery: injury & remodeling studies. ISRN Minim. Invasive Surg. 2013, 1–10. 10.1155/2013/167407 PMC3677797 23762781

[B11] Jacobs M. Forst C. (1981). Toxicological effects of sodium selenite in Sprague-Dawley rats. J. Toxicol. Environ. Health 8 (4), 575–585. 10.1080/15287398109530092 7338930

[B12] Jochen D. Jan-Marcus D. Johann B. Denise H. K. Sedding D. G. (2015). Emerging translational approaches to target STAT3 signalling and its impact on vascular disease. Cardiovasc. Res. 106 (3), 365–374. 10.1093/cvr/cvv103 25784694 PMC4431663

[B13] Kim J. Ko J. (2014). Human sLZIP promotes atherosclerosis via MMP-9 transcription and vascular smooth muscle cell migration. FASEB J. 28 (11), 5010–5021. 10.1096/fj.14-259218 25077563

[B14] Kim J. B. Yang E. Y. Woo J. Kwon H. Lim W. Moon B. I. (2020). Sodium selenite enhanced the anti-proliferative effect of MEK-ERK inhibitor in thyroid cancer cells. In Vivo 34 (1), 185–190. 10.21873/invivo.11760 31882478 PMC6984107

[B15] Kim Y. H. Lee S. J. Seo K. W. Bae J. U. Park S. Y. Kim E. K. (2013). PAF enhances MMP-2 production in rat aortic VSMCs via a β-arrestin2-dependent ERK signaling pathway. J. Lipid. Res. 54 (10), 2678–2686. 10.1194/jlr.M037176 23911909 PMC3770081

[B16] Lacolley P. Regnault V. Segers P. Laurent S. (2017). Vascular smooth muscle cells and arterial stiffening: relevance in development, aging, and disease. Physiol. Rev. 97 (4), 1555–1617. 10.1152/physrev.00003.2017 28954852

[B17] Laureano-Melo R. Império G. E. da Silva-Almeida C. Kluck G. E. G. Cruz Seara F. de. A. da Rocha F. F. (2015). Sodium selenite supplementation during pregnancy and lactation promotes anxiolysis and improves mnemonic performance in wistar rats' offspring. Pharmacol. Biochem. Behav. 138, 123–132. 10.1016/j.pbb.2015.09.007 26364924

[B18] Li S. Zhai C. Shi W. Feng W. Xie X. Pan Y. (2020). Leukotriene B4 induces proliferation of rat pulmonary arterial smooth muscle cells via modulating GSK-3β/β-catenin pathway. Eur. J. Pharmacol. 867, 172823. 10.1016/j.ejphar.2019.172823 31770525

[B19] Luo H. Yang Y. Duan J. Wu P. Jiang Q. Xu C. (2013). PTEN-regulated AKT/FoxO3a/Bim signaling contributes to reactive oxygen species-mediated apoptosis in selenite-treated colorectal cancer cells. Cell Death Dis. 4 (2), e481. 10.1038/cddis.2013.3 23392169 PMC3734838

[B20] Muto A. Fitzgerald T. N. Pimiento J. M. Maloney S. P. Teso D. Paszkowiak J. J. (2007). Smooth muscle cell signal transduction: implications of vascular biology for vascular surgeons. J. Vasc. Surg. 45 Suppl A (6S), A15–A24. 10.1016/j.jvs.2007.02.061 17544020 PMC1939976

[B21] Okuno T. Honda E. Arakawa T. Ogino H. Ueno H. (2014). Glutathione-dependent cell cycle G1 arrest and apoptosis induction in human lung cancer A549 cells caused by methylseleninic acid: comparison with sodium selenite. Biol. Pharm. Bull. 37 (11), 1831–1837. 10.1248/bpb.b14-00453 25177040

[B22] Orozco L. D. Liu H. Chen B. B. Buciuc R. F. Fratkin J. D. Pisarello J. C. (2012). Aortic response to balloon injury in obese zucker rats. Comp. Med. 62 (4), 264–270. 10.1111/j.1439-0531.2012.02104.x 23043778 PMC3415367

[B23] Pfister C. Dawzcynski H. Schingale F. J. (2016). Sodium selenite and cancer related lymphedema: biological and pharmacological effects. J. Trace Elem. Med. Biol. 37, 111–116. 10.1016/j.jtemb.2016.05.005 27267968

[B24] Rabinovitch M. (2012). Molecular pathogenesis of pulmonary arterial hypertension. J. Clin. Invest. 122 (12), 4306–4313. 10.1172/JCI60658 23202738 PMC3533531

[B25] Rayman M. P. (2008). Food-chain selenium and human health: emphasis on intake. Br. J. Nutr. 100 (2), 254–268. 10.1017/S0007114508939830 18346308

[B26] Ren Y. Huang F. Liu Y. Yang Y. Jiang Q. Xu C. (2009). Autophagy inhibition through PI3K/Akt increases apoptosis by sodium selenite in NB4 cells. BMB Rep. 42 (9), 599–604. 10.5483/BMBRep.2009.42.9.599 19788862

[B27] Rensen S. S. M. Doevendans P. A. F. M. van Eys G. J. J. M. (2007). Regulation and characteristics of vascular smooth muscle cell phenotypic diversity. Neth. Heart J. 15 (3), 100–108. 10.1007/BF03085963 17612668 PMC1847757

[B28] Riascos-Bernal D. F. Chinnasamy P. Gross J. N. Almonte V. Egaña-Gorroño L. Parikh D. (2017). Inhibition of smooth muscle β-catenin hinders neointima formation after vascular injury. Arterioscler. Thromb. Vasc. Biol. 37 (5), 879–888. 10.1161/ATVBAHA.116.308643 28302627 PMC5408313

[B29] Shigemi Z. Manabe K. Hara N. Baba Y. Hosokawa K. Kagawa H. (2017). Methylseleninic acid and sodium selenite induce severe ER stress and subsequent apoptosis through UPR activation in PEL cells. Chem. Biol. Interact. 266, 28–37. 10.1016/j.cbi.2017.01.027 28161410

[B30] Soliman S. M. Wadie W. Shouman S. A. Ainshoka A. A. (2018). Sodium selenite ameliorates both intestinal and extra-intestinal changes in acetic acid-induced colitis in rats. Naunyn Schmiedebergs Arch. Pharmacol. 391 (6), 639–647. 10.1007/s00210-018-1491-7 29656366

[B31] Sprague A. H. Khalil R. A. (2009). Inflammatory cytokines in vascular dysfunction and vascular disease. Biochem. Pharmacol. 78 (6), 539–552. 10.1016/j.bcp.2009.04.029 19413999 PMC2730638

[B32] Stone G. W. Midei M. Newman W. Sanz M. Hermiller J. B. Williams J. (2008). Comparison of an everolimus-eluting stent and a paclitaxel-eluting stent in patients with coronary artery disease: a randomized trial. JAMA 299 (16), 1903–1913. 10.1001/jama.299.16.1903 18430909

[B33] Tao Z. Gan Z. Xinying Z. Kangfeng J. Haichong W. Ganzhen D. (2018). Sodium selenite induces apoptosis via ROS-mediated NF-κB signaling and activation of the Bax-caspase-9-caspase-3 axis in 4T1 cells. J. Cell. Physiol. 234 (3), 2511–2522. 10.1002/jcp.26783 30218457

[B34] Thompson A. A. Lawrie A. (2017). Targeting vascular remodeling to treat pulmonary arterial hypertension. Trends Mol. Med. 23 (1), 31–45. 10.1016/j.molmed.2016.11.005 27989641

[B35] Wu H. Cheng X. W. Hu L. Takeshita K. Hu C. Du Q. (2016). Cathepsin S activity controls injury-related vascular repair in mice via the TLR2-mediated p38MAPK and PI3K-Akt/p-HDAC6 signaling pathway. Arterioscler. Thromb. Vasc. Biol. 36 (8), 1549–1557. 10.1161/ATVBAHA.115.307110 27365406 PMC4961274

[B36] Wu X. Zhao G. He Y. Wang W. Yang C. S. Zhang J. (2019). Pharmacological mechanisms of the anticancer action of sodium selenite against peritoneal cancer in mice. Pharmacol. Res. 147, 104360. 10.1016/j.phrs.2019.104360 31326526

[B37] Wu Y. Cai C. Yang L. Xiang Y. Zhao H. Zeng C. (2020). Inhibitory effects of formononetin on the monocrotalineinduced—pulmonary arterial hypertension in rats. Mol. Med. Rep. 21 (3), 1192–1200. 10.3892/mmr.2020.10911 31922224 PMC7003019

[B38] Xu T. Zhu H. Li D. Lang Y. Cao L. Liu Y. (2015). Luteolin inhibits angiotensin II-stimulated VSMC proliferation and migration through downregulation of akt phosphorylation. Evid. Based Complement Alternat. Med. 2015, 931782. . 10.1155/2015/931782 26347796 PMC4546982

[B39] Xu X. L. Ling D. Y. Zhu Q. Y. Fan W. J. Zhang W. (2013). The effect of 2,3,4',5-tetrahydroxystilbene-2-0-β-D glucoside on neointima formation in a rat artery balloon injury model and its possible mechanisms. Eur. J. Pharmacol. 698 (1-3), 370–378. 10.1016/j.ejphar.2012.11.019 23178522

[B40] Zhu N. Xiang Y. Zhao X. Cai C. Chen H. Jiang W. (2019). Thymoquinone suppresses platelet-derived growth factor-BB-induced vascular smooth muscle cell proliferation, migration and neointimal formation. J. Cell. Mol. Med. 23 (12), 8482–8492. 10.1111/jcmm.14738 31638340 PMC6850929

[B41] Zhu N. Zhao X. Xiang Y. Ye S. Huang J. Hu W. (2016). Thymoquinone attenuates monocrotaline-induced pulmonary artery hypertension via inhibiting pulmonary arterial remodeling in rats. Int. J. Cardiol. 221, 587–596. 10.1016/j.ijcard.2016.06.192 27420584

